# Digital practical course of otorhinolaryngology and examination technique “to go”

**DOI:** 10.3205/zma001360

**Published:** 2020-12-03

**Authors:** Stefan Kaulitz, Jonas Engert, Carolin Roos, Maike Filsinger, Sarah König, Stephan Hackenberg

**Affiliations:** 1 Universitätsklinikum Würzburg, Klinik und Poliklinik für Hals-, Nasen- und Ohrenkrankheiten, plastische und ästhetische Operationen, Würzburg, Germany; 2 Universitätsklinikum Würzburg, Institut für Medizinische Lehre und Ausbildungsforschung, Würzburg, Germany

**Keywords:** ENT, digital teaching, examination practical, propaedeutics, tutorial

## Abstract

**Objective: **The development of presentation-free teaching formats for practical exercises is a particular challenge. The objective of the presented project was the implementation of the practical course in otorhinolaryngology for learning examination techniques through digital distance learning.

**Methods:** Disposable instruments for a comprehensive otorhinolaryngologic examination were purchased and made available to the students. The necessary light sources were everyday sports headlamps or flashlight constructions. The theoretical basics were taught using the learning platform CaseTrain, including photographic, audio and video material. Corresponding success controls in the sense of a digital short test were integrated here. In addition, video tutorials were used to present the examinations to be imitated in detail. In order to increase motivation, a creative photo competition was also announced, in which the practical tasks that were finally carried out independently were checked.

**Results: **All students of the semester received a set of instruments for independent practical training. The entrance exam was passed by all students, and at least one photo was received from all students, many of which were particularly creative in the competition.

**Conclusion: **The presented concept is a possibility to convert practical teaching formats into a digital presence-free concept. It included the teaching and testing of basic knowledge, instructions for practical exercises, the independent performance of subject-specific examination techniques and an increase in motivation and simultaneous control through gamification. We consider this teaching principle to be an attractive option for future “Flipped Classroom” concepts with a gradual resumption of face-to-face teaching.

## Zusammenfassung

**Zielsetzung: **Die Entwicklung präsenzfreier Lehrformate für praktische Übungen stellt eine besondere Herausforderung dar. Zielsetzung des vorgestellten Projekts war die Umsetzung des Praktikums der Hals-Nasen-Ohren-Heilkunde zum Erlernen von Untersuchungstechniken durch digitale Fernlehre.

**Methodik: **Es wurde kostengünstig fachspezifisches Einmal-Instrumentarium für eine umfassende hals-nasen-ohren-ärztliche Spiegeluntersuchung angeschafft, welches den Studierenden zur Verfügung gestellt wurde. Als erforderliche Lichtquellen dienten alltagsgebräuchliche Sportstirnlampen oder Taschenlampenkonstruktionen. Die theoretischen Grundlagen wurden mittels der Lernplattform CaseTrain unter Einbeziehung von Foto-, Audio- und Videomaterial vermittelt. Entsprechende Erfolgskontrollen im Sinne eines digitalen Kurztestates wurden hier integriert. Des Weiteren dienten Video-Tutorials der genauen Darstellung der nachzuahmenden Untersuchungen. Zur Steigerung der Motivation wurde zudem ein kreativer Fotowettbewerb ausgeschrieben, bei dem die abschließend eigenständig durchgeführten praktischen Aufgaben kontrolliert wurden.

**Ergebnisse:** Sämtliche Studierende des Semesters erhielten ein Instrumentenset zur eigenständigen Praktikumsdurchführung. Das Eingangstestat wurde von allen Studierenden bestanden, ebenso ging von allen Studierenden mindestens ein Foto ein, viele gestalteten dieses im Rahmen des Wettbewerbs besonders kreativ.

**Schlussfolgerung: **Das vorgestellte Konzept stellt eine Möglichkeit zur Umsetzung praktischer Lehrformate auf ein digitales präsenzfreies Konzept dar. Es beinhaltete eine Vermittlung und Überprüfung von Grundlagenwissen, Anleitungen zu praktischen Übungen, die eigenständige Durchführung fachspezifischer Untersuchungstechniken und eine Motivationssteigerung und gleichzeitige Kontrolle durch Gamification. Wir erachten dieses Lehrprinzip als attraktive Option für zukünftige „Flipped Classroom“ Konzepte bei schrittweiser Wiederaufnahme von Präsenzlehre.

## 1. Introduction

The SARS-CoV 2 pandemic has challenged medical teaching, but also left it highly open to new development opportunities [1], [2]. In particular, teaching formats that include practical exercises were difficult to replace by digital distance learning and corresponding examinations [3]. In the following article we would like to present a concept for the conversion of a practical course for learning ENT-examination techniques with an upstream entrance exam.

## 2. Project description

**Figure 1:**
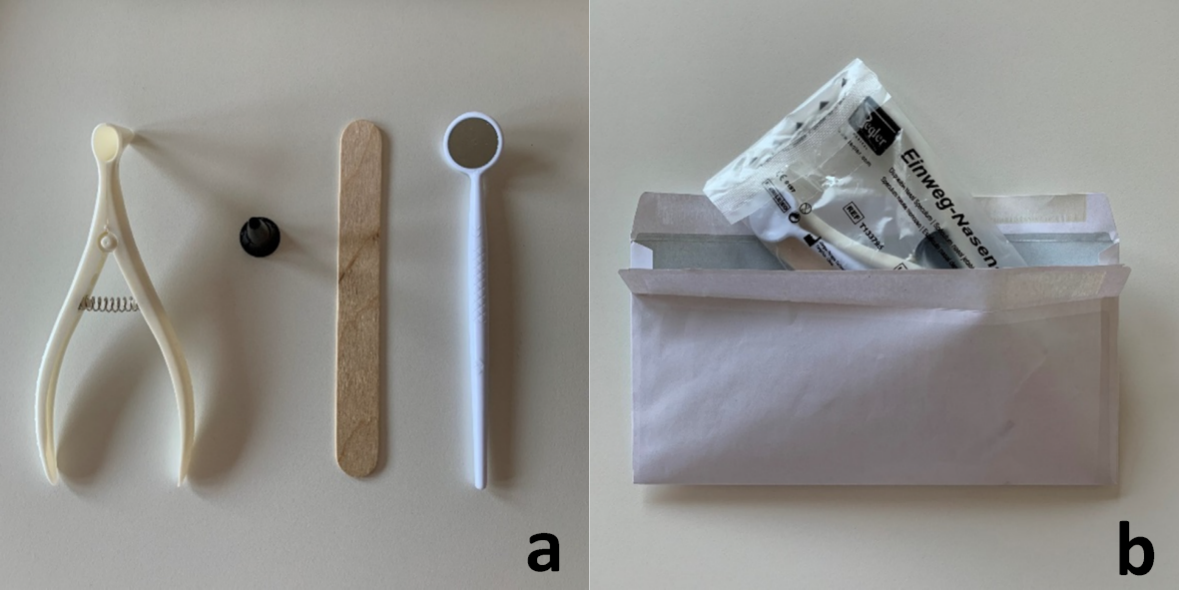
Examination kit for learning a comprehensive otorhinolaryngologic mirror examination in the context of digital distance learning - The examination kit contained the following disposable instruments: a nasal speculum, an ear speculum, a mouth spatula and a laryngeal mirror (a). The instruments could be picked up personally or delivered by mail (b).

**Figure 2:**
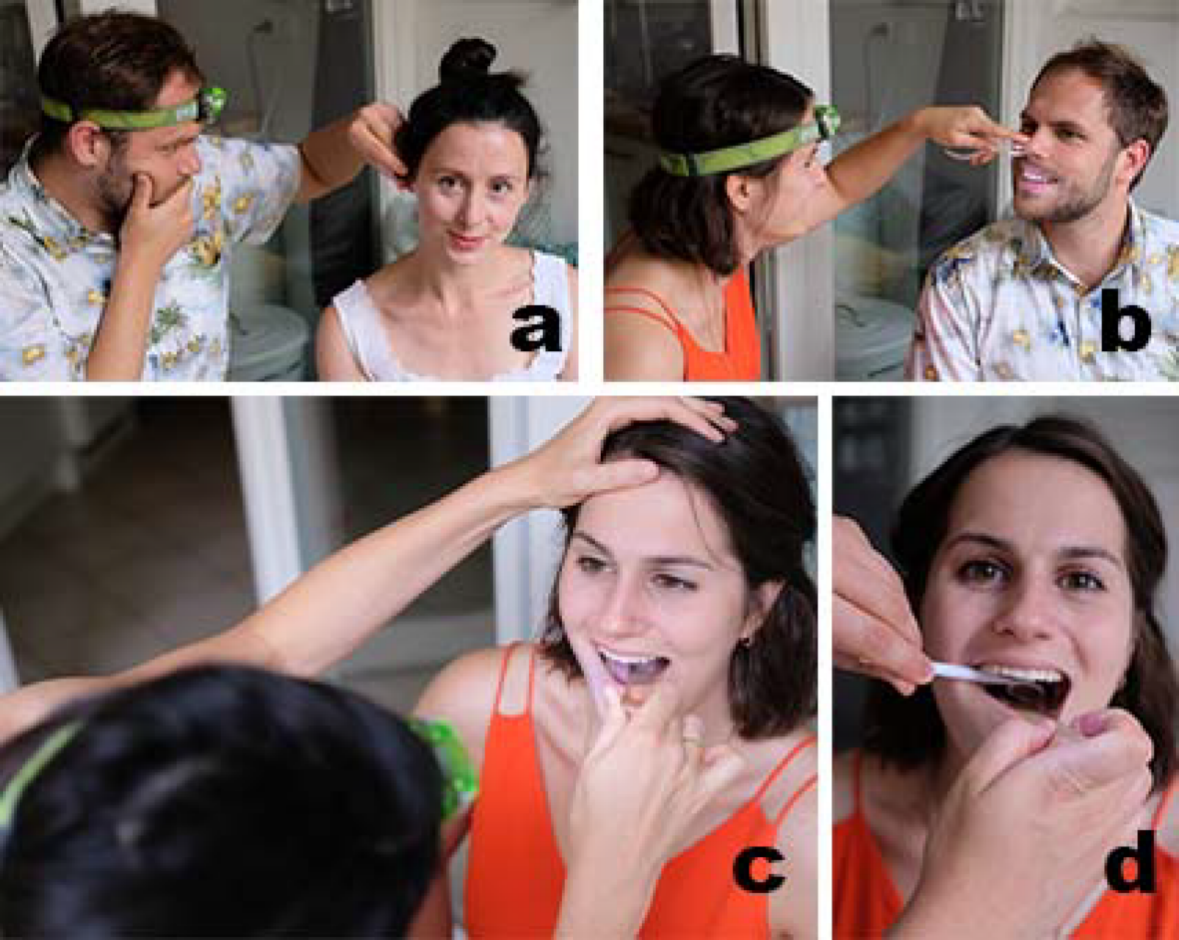
Exemplary performance of a mirror examination of ENT-experienced PJ students with the examination kit - By using an external light source (e.g. headlamp), the examination kit enabled a professional otoscopy (a), anterior rhinoscopy (b), oral cavity inspection (c) and indirect laryngoscopy (d) to be performed. The independent performance of the examination methods was achieved by imitating the video tutorials provided.

Due to the strict contact restrictions in Bavaria, the ENT practical course as part of the curricular teaching was excluded as a classroom event. The examinations in the field of otorhinolaryngology require special instruments as well as a person to be examined (e.g. peer). Disposable instruments were procured via online trading and an examination kit was compiled for the students. It included a nasal speculum, an ear speculum, a mouth spatula and a laryngeal mirror (cost: 1 €/student; see figure 1 (Fig. 1), point a). The students could use existing headlamps or flashlight constructions as the required light source (see figure 2 (Fig. 2)).

The teaching unit started with an introductory information video about the structure and procedures of the digital lab.

**Figure 3:**
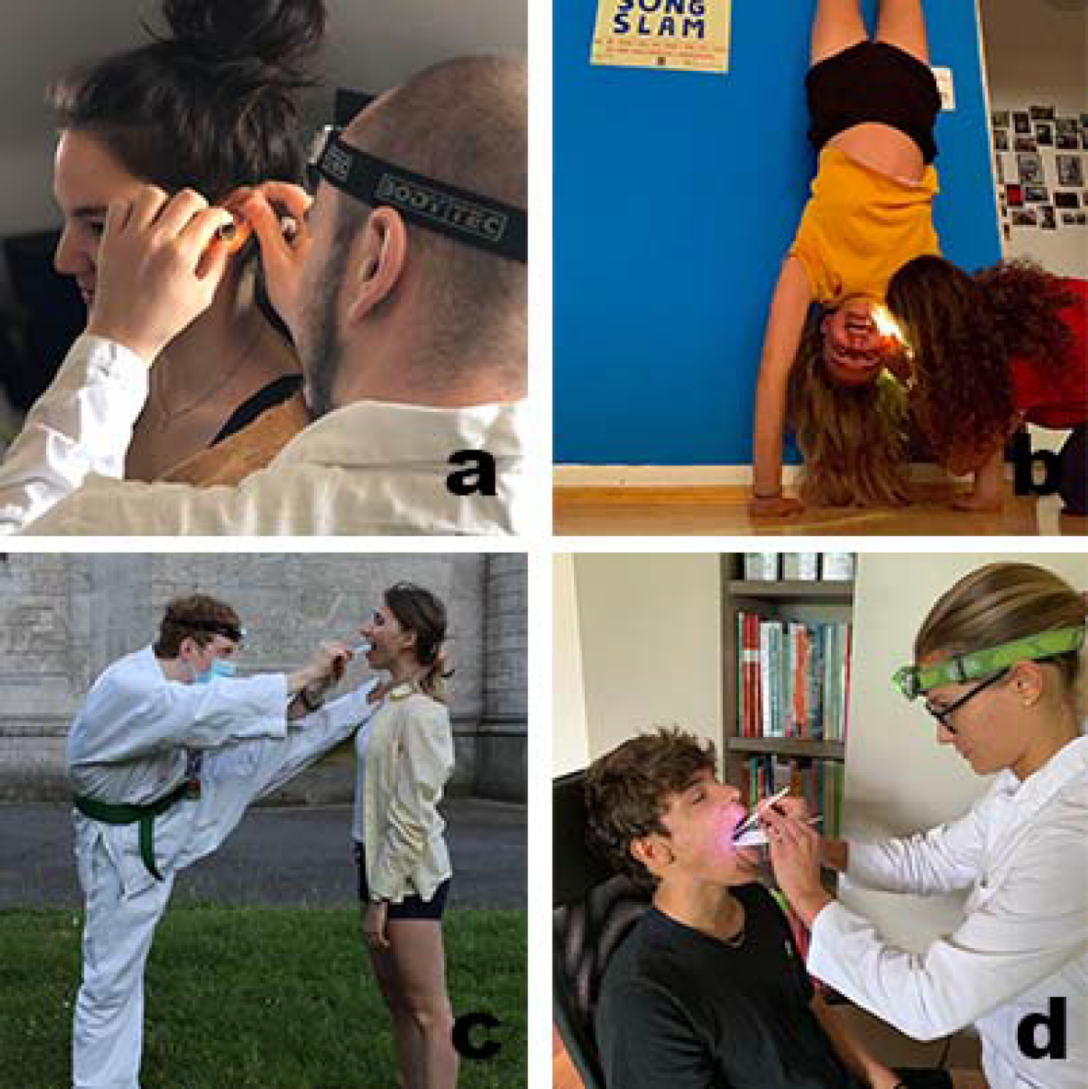
Examples of the contributions submitted by the students in the photo competition - Gamification was used to control the implementation of the examination methods. Otoscopy (a+b), oral inspection and indirect laryngoscopy (c+d) were presented in different ways. This method also served to increase the motivation of the students, who provided proof of work either in the most precise (a+d) or creative (b+c) way.

The actual propaedeutics lecture and the otherwise orally tested entrance exam were replaced by e-learning cases using the learning platform “CaseTrain” [4]. This was originally developed for practical patient cases with integrated test results. Photo, audio and video material can be integrated. The interdisciplinary propaedeutic cases developed by lecturers, physicians and advanced students (practical year) contained ten test questions (pass mark 60%) on basic ENT knowledge and examination techniques. Specially created examination videos (tutorials) served the students as detailed preparation for the ENT examination, which they had to perform independently. In order to increase the motivation for learning in asynchronous teaching, a photo competition was announced, which at the same time served as a control for the practical tasks (see figure 3 (Fig. 3)). For this purpose, only pictures for which a written declaration of consent was available were used (voluntary). Possible problems that could not be solved during the examination exercises were dealt with in subsequent video conferences.

## 3. Results

The examination kits were picked up personally by almost all students at the clinic's teaching office. A few students who spent the semester outside of Würzburg either took care of the examination material themselves or received the kits by mail (see figure 1 (Fig. 1), point b).

**Figure 4:**
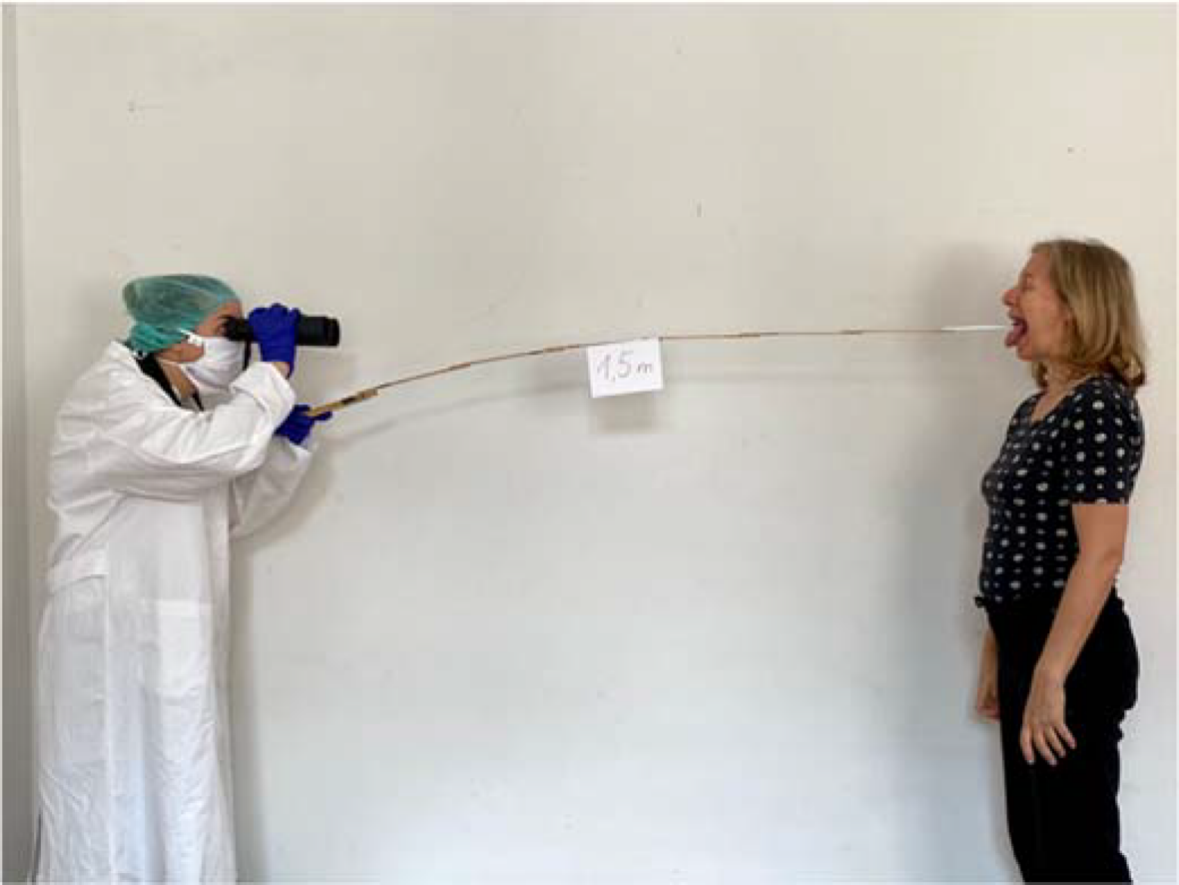
Winner of the photo competition - Due to the creative realization of the work order in combination with the allusion to the SARS-CoV-2 pandemic and the required minimum distance, the interprofessional jury decided in favor of this photo.

The entrance examination was passed by all students. A photograph of the examination exercises was also received from all students. Figure 3 (Fig. 3) and figure 4 (Fig. 4) show some implementations in the students' own design environment.

## 4. Discussion

Teaching concepts based on e-learning for ENT practical courses are rare, although the need to establish digital teaching formats has already been addressed at German ENT university hospitals for both student teaching [5] and specialist training [6]. 

The declared goal of the project was to develop a teaching and examination concept for the time without classroom teaching. The format of the e-learning cases, which was evaluated by students as extremely effective and useful with regard to “blended learning” [7], [8], in combination with the provision of one-time instruments, made it possible to implement the above-mentioned aspects simultaneously.

The developed format should include

- teaching and testing of basic knowledge,
- instructions for practical exercises (model learning via video tutorials),
- independent execution of the exercises by imitation,
- motivation and implementation control through gamification (photo competition).

In addition, it can provide the basis for future teaching according to the principle of the “flipped classroom”, if face-to-face teaching in a hybrid version can be resumed with a sense of proportion and sharing during the low-prevalence phase of SARS-CoV-2. The “Flipped Classroom” integrates didactic content more effectively into the everyday medical life of students and teachers [9].

Video demonstrations can be used efficiently for learning practical medical skills [10], [11]. Studies that compare learning an ENT examination by face-to-face teaching with digital teaching have not been described in the literature so far. A study with Canadian medical students on the management of epistaxis showed a significantly better performance of a computer-based teaching module compared to text-based methods [12]. A comparison of knowledge transfer on ENT emergencies between traditional face-to-face teaching and e-learning based methods showed no significant difference in quality [13]. These data show that teaching clinical skills can also be sensibly done digitally. If face-to-face teaching is resumed, the CaseTrains presented here, including preliminary exercises of examination techniques in connection with the subsequent practical training in otorhinolaryngology as a “Flipped Classroom”, could exploit new potentials [14].

Digital teaching content is the mainstay in the teaching of theoretical fundamentals, on the basis of which problem-oriented and case-based face-to-face sessions are then developed [15]. In the future, the effectiveness of this teaching must be evaluated on the basis of the examination results and the practical competence (e.g. OSCE) of the students.

The choice to motivate through gamification (here photo competition) and to control the execution of the practical exercises proved itself and corresponded with the experiences reported in the literature [16].

## Competing interests

The authors declare that they have no competing interests.
